# Supplementary material for: Electrochemical Synthesis of Nb-Doped BaTiO3 Nanoparticles with Titanium-Niobium Alloy as Electrode
Source: Nanomaterials (Basel). 2023 Jan 6;13(2):252. doi: 10.3390/nano13020252 (PMC9862403; doi:10.3390/nano13020252)
Supplement: Supplementary file 1 [file nanomaterials-13-00252-s001.zip › nanomaterials-2083484-supplementary.docx]

Electrochemical Synthesis of Nb-doped BaTiO_3_ Nanoparticles with Titanium-Niobium Alloy as Electrode

Qi Yuan ^1^, Wencai Hu ^2^, Tao Wang ^3^, Sen Wang ^1,^*, Gaobin Liu ^1^, Xueyan Han ^1^, Feixiang Guo ^1^ and Yongheng Fan ^1^

^1^ School of Materials and Metallurgy, University of Science and Technology Liaoning, Anshan 114051, China; yq15041841142@126.com (Q.Y.); gbliu@ustl.edu.cn (G.L.); hanxyyan@163.com (X.H.); [gfx729259022@163.com](mailto:gfx729259022@163.com) (F.G.); 202085600375@stu.ustl.edu.cn (Y.F.)

^2^ Fujian Huaqing Electronic Material Technology Co., Ltd., Quanzhou, Fujian 362000, China; hwc0708@163.com

^3^ Jiangsu Can Qin Technology Co., Ltd., Suzhou ,Jiangsu 215633, China; wangtao6769@gmail.com

***** Correspondence: wsenl@yeah.net

**SAXS data**


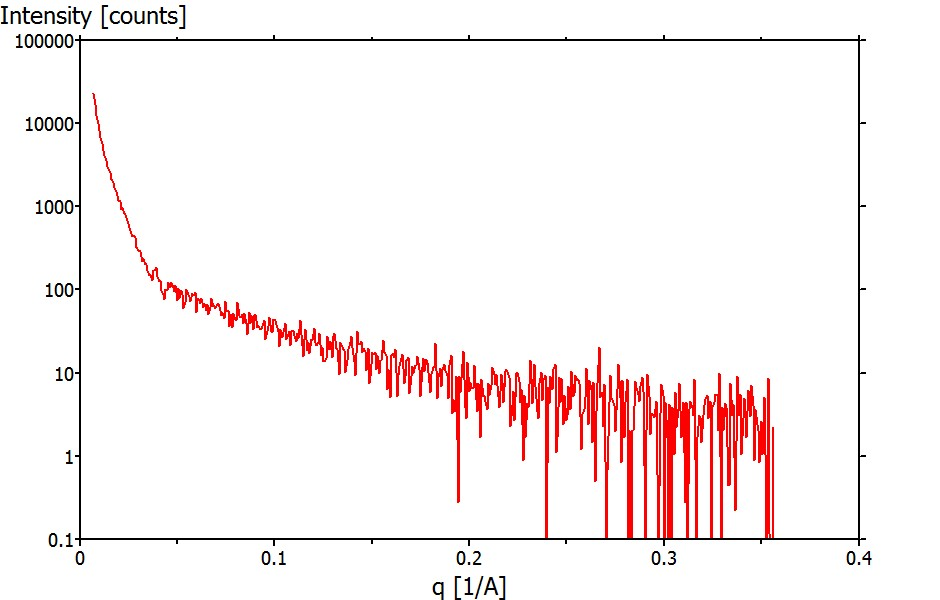


**Figure S1.** SAXS data (NaOH 1.0 mol·L^-1^)


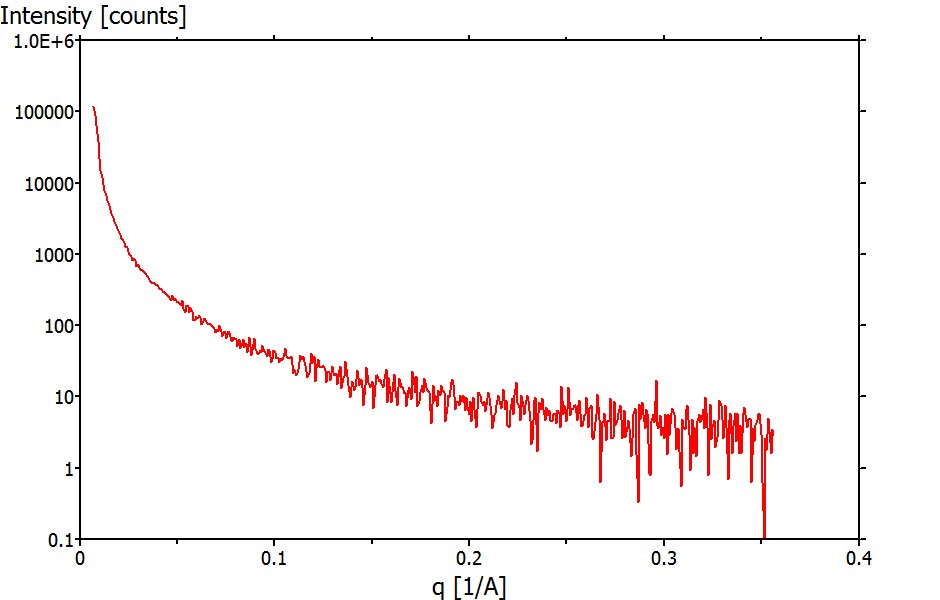


**Figure S2.** SAXS data (NaOH 1.5 mol·L^-1^)


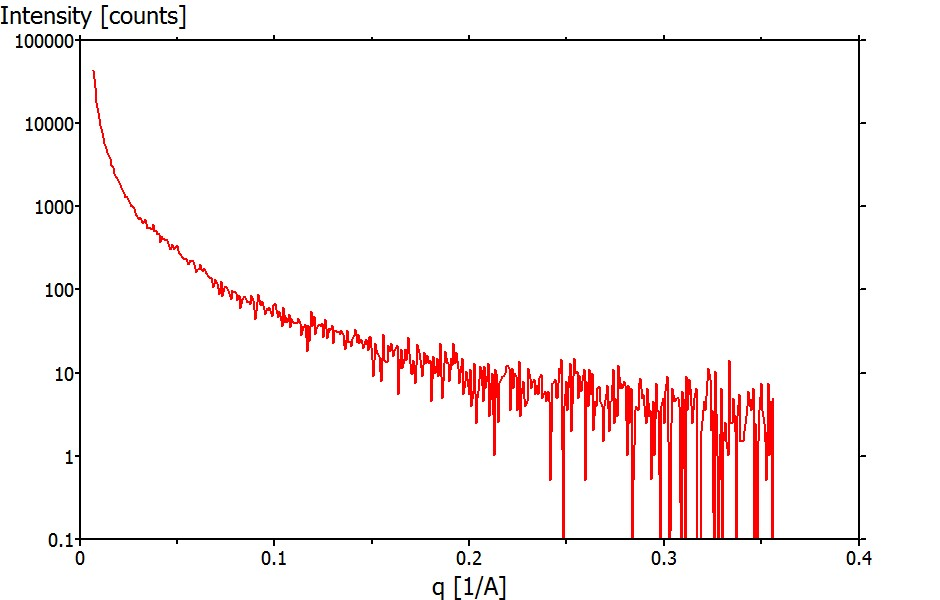


**Figure S3.** SAXS data (NaOH 2.0 mol·L^-1^)


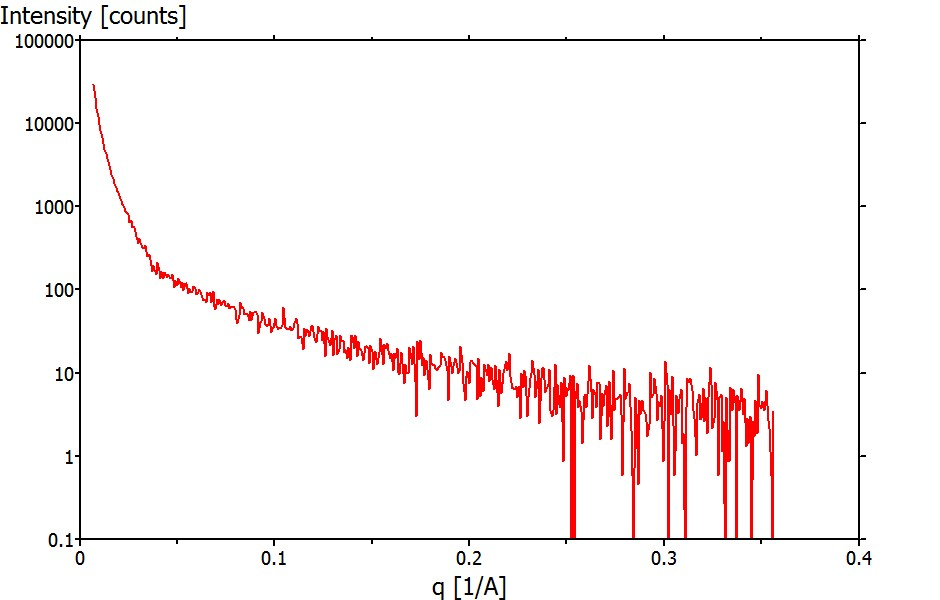


**Figure S4.** SAXS data (NaOH 2.5 mol·L^-1^)


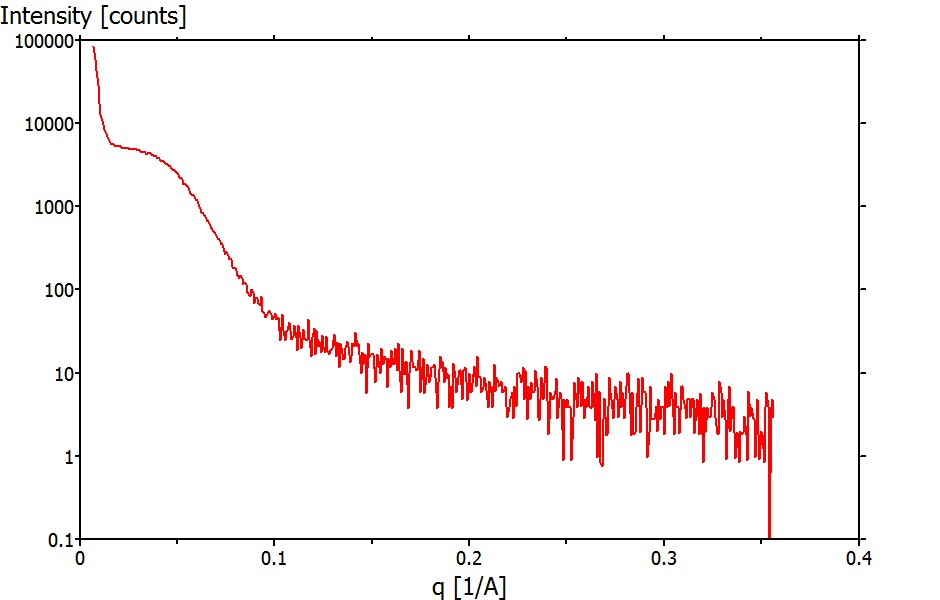


**Figure S5.** SAXS data (NaOH 3.0 mol·L^-1^)
